# The effect of brief pre-anesthetic exercise therapy of jaw and neck joints on mouth opening, neck extension, and intubation conditions during induction of general anesthesia: a randomized controlled trial

**DOI:** 10.1186/s12871-020-0939-8

**Published:** 2020-01-29

**Authors:** Sue Young Lee, Sung Il Bae, Sang-Hwan Do, Ju-Tae Sohn, Jin-Woo Park

**Affiliations:** 1 Department of Anesthesiology and Pain Medicine, ThanQ Seoul Thyroid-Head & Neck Surgery Center, Seoul, South Korea; 2 Department of Anesthesiology and Pain Medicine, Jinju Gyeongsang National University Hospital, Jinju, South Korea; 3 Department of Anesthesiology and Pain Medicine, Seoul National University Bundang Hospital, 82 Gumi-ro, 173 Beon-gil, Bundang-gu, Seongnam, Gyeonggi-do 13620 Republic of Korea; 4 Department of Anesthesiology and Pain Medicine, Seoul National University College of Medicine, Seoul, South Korea; 5 Department of Anesthesiology and Pain Medicine, Gyeongsang National University College of Medicine, Institute of Health Sciences, Gyeongsang National University, Jinju, South Korea

**Keywords:** Anesthesia, general, Exercise, Intubation, Mouth opening, Neck extension

## Abstract

**Background:**

The effort to improve tracheal intubation process is clinically valuable. We hypothesized that a preoperative brief exercise therapy would increase mouth opening and neck extension, enhancing intubation conditions during general anesthesia.

**Methods:**

Patients undergoing general anesthesia were randomized into two groups. The exercise group performed the exercise regimen including masseter muscle massage and stretching of jaw and neck joints before anesthetic induction, while the control did not. Before (baseline) and after the intervention, we evaluated Mallampati score, mouth aperture size, and sternomental distance. After tracheal intubation, intubation difficulty scale with direct laryngoscope and oropharyngeal soft tissue injury were also evaluated.

**Results:**

A total of 138 patients completed the analysis (control = 68, exercise = 70). Baseline characteristics did not differ between groups. At anesthetic induction, there was a significant difference in Mallampati score between the two groups (*P* = 0.039) and the incidence of Mallampati scores of 1 was higher in the exercise group (odds ratio [95% CI]: 2.1 [1.0–4.3], *P* = 0.043). Mouth opening after the intervention was greater in the exercise group than in the control group (estimated difference [95% CI]: − 2.4 [− 4.8 – -0.1], *P* = 0.042) and sternomental distance was similar between the two groups (estimated difference [95% CI]: − 3.7 [− 9.0–1.7, *P* = 0.175). The exercise group showed less soft tissue injuries (odds ratio [95% CI]: 0.2 [0.1–0.8], *P* = 0.009), however, intubation difficulty scale did not differ between the study groups (*P* = 0.112).

**Conclusions:**

The brief pre-anesthetic exercise improved intubation conditions and enabled faster tracheal intubation with less injury to oropharyngeal soft tissue.

**Trial registration:**

Clinical Research Information Service (registration number: KCT0002618), registered at December 28, 2017.

## Background

Limited neck movement and a short inter-incisor gap may interfere with exposure of the larynx during direct laryngoscopy and are significant risk factors for difficult tracheal intubation [1–5]. When glottic exposure is inadequate with a direct laryngoscope, other instruments such as intubation stylet, video laryngoscope, or fiber-optic bronchoscope can be utilized to access the larynx [6, 7]. However, difficult intubation cannot be exactly predicted in advance [3, 5, 8]. Complications such as soft tissue injuries and dental trauma may also occur during successful intubation [9, 10]. Without a doubt, improvement of intubation conditions is of clinical significance for patients undergoing general anesthesia.

Various exercise regimens based on stretching and muscle massage have been proposed to treat patients with temporomandibular joint disorders and impaired mouth opening [11]. Furthermore, previous studies demonstrated that brief stretching or massage of joint muscles could increase range of motion and decrease muscle stiffness in healthy volunteers as well as subjects with musculoskeletal abnormalities [12–18]. Therefore, we hypothesized that pre-anesthetic exercise therapy of jaw (temporomandibular) and neck (cervical vertebral) joints could enhance mouth opening and neck mobility during anesthetic induction and facilitate the process of orotracheal intubation.

We performed a randomized controlled trial to evaluate the effects of a single brief pre-anesthetic exercise therapy session on mouth opening, neck extension, intubation difficulty, and soft tissue injuries to the oropharyngeal area in patients undergoing general anesthesia.

## Methods

### Study

The protocol for this prospective multicenter trial was approved by the Institutional Review Boards of Seoul National University Bundang Hospital (study number: B-1709-423-307) and Jinju Gyeongsang National University Hospital (study number: GNUH 2018–05–019-001). It was registered at the Clinical Research Information Service (https://cris.nih.go.kr; registration number: KCT0002618; date of registration: December 28, 2017). Written informed consent was obtained from all patients before surgery. Patients were enrolled from January to October 2018 at both institutions. This study adhered to CONSORT guidelines.

### Patients

Adult patients with American Society of Anesthesiologists physical status I–II, aged 20–70 years, who were scheduled to undergo elective surgery under general anesthesia were enrolled in this study. We excluded patients who were likely to encounter difficulties or pain when actively performing the jaw and neck exercises: Patients with cognitive disorders, temporomandibular joint disorders, or cervical spine diseases. For safety reasons, we excluded patients under consideration for awake intubation using a fiber-optic bronchoscope, such as those with craniofacial anomaly or mechanical airway obstruction. Patients with tracheostomy or who were to receive oropharyngeal surgery during this protocol were also excluded. Enrolled patients were randomly allocated to either the exercise or the control group, using a computer-generated randomization code (Random Allocation Software Version 1.0; University of Medical Sciences, Isfahan, Iran). The allocation ratio was 1:1. Randomization was performed by an independent anesthesiologist in each hospital. These individuals were only involved in patient allocation and coaching of the exercise, and not in any aspects of anesthesia, data analysis or interpretation.

### Intervention, anesthesia, and study outcomes

**Fig. 1:**
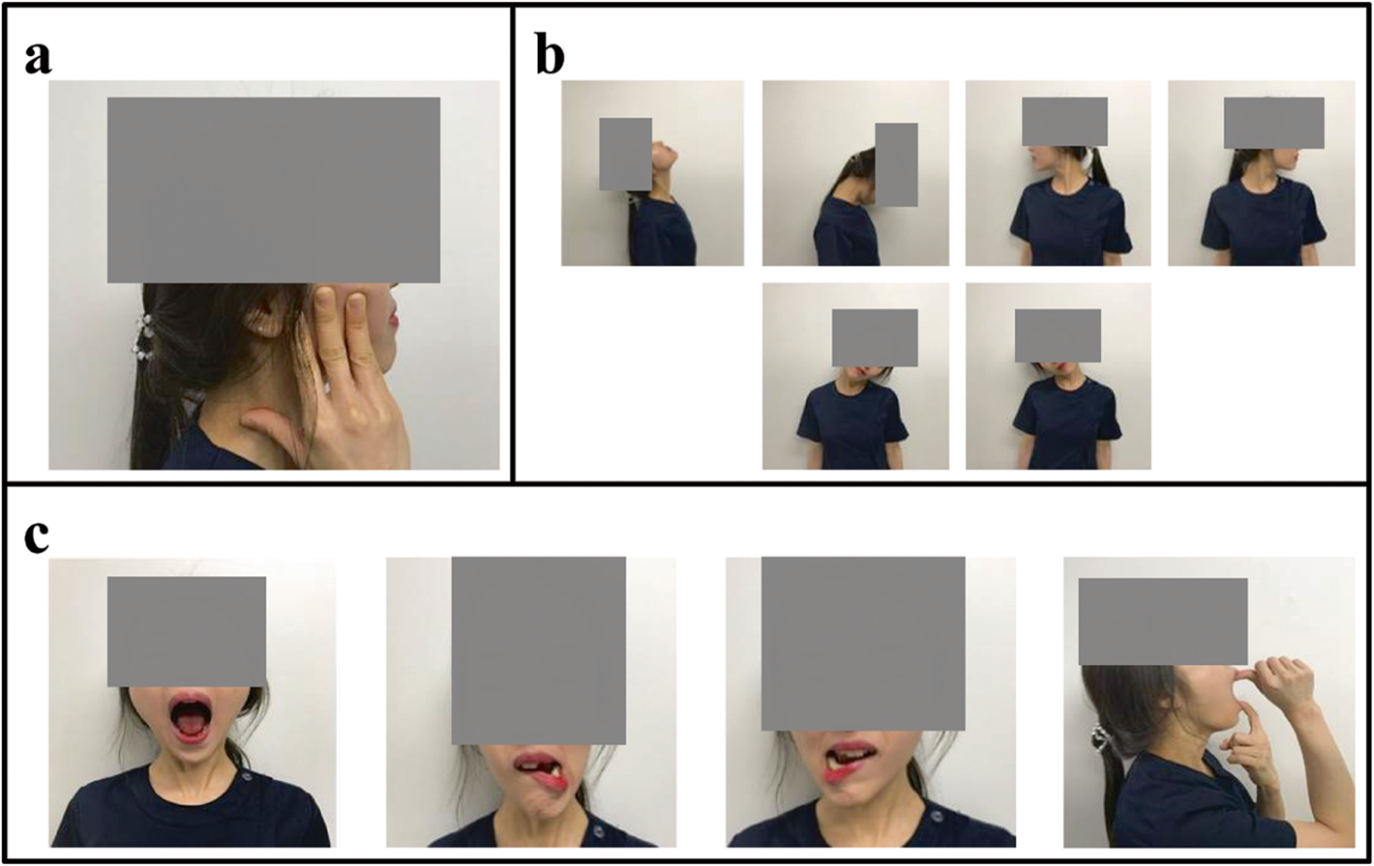
The exercise regimen on jaw and neck joints (Exercise sequence: a – b – c – a). **a** Manual massage of masseter muscle (30 s); **b** Extension, flexion, rotation, and lateral flexion of neck (2 min); **c** Active and passive maximum mandibular opening and lateral deviation of temoporomandibular joint (2 min)

At the reception area, each patient’s basal characteristics related to tracheal intubation difficulty were assessed [2–5]. These included mouth aperture size defined as the maximum interincisal distance, Mallampati score, sternomental distance (SMD) as an indicator of neck mobility, thyromental distance, and the presence of buck teeth, were assessed by an anesthesiology resident in each hospital who only participated in these examinations and did not know the patient group. We used a single assessor in each hospital, to minimize inter-rater bias. In this protocol, Mallampati scoring was performed in sitting position, with the tongue fully protruded, and without phonation [19]. During the measurements, the sitting patients were encouraged to open their mouths as wide as possible and to fully extend their head on the neck. After the baseline examination, patients in the exercise group were guided to perform exercise therapy for 5 min at the reception area, before transferral to the operating room. This brief exercise regimen comprises masseter muscle massage and active/passive stretching of the jaw and neck joints (Fig. 1). Stretching was performed to the maximum range of motion within the level not causing pain. The patients in the control group waited at the reception area, without any exercise, before transfer to the operating room. No pre-medication was given to patients in either group. Patients were instructed not to reveal their group information to anyone after the intervention. The importance of blindness was emphasized repeatedly to all patients throughout the protocol.

**Fig. 2:**
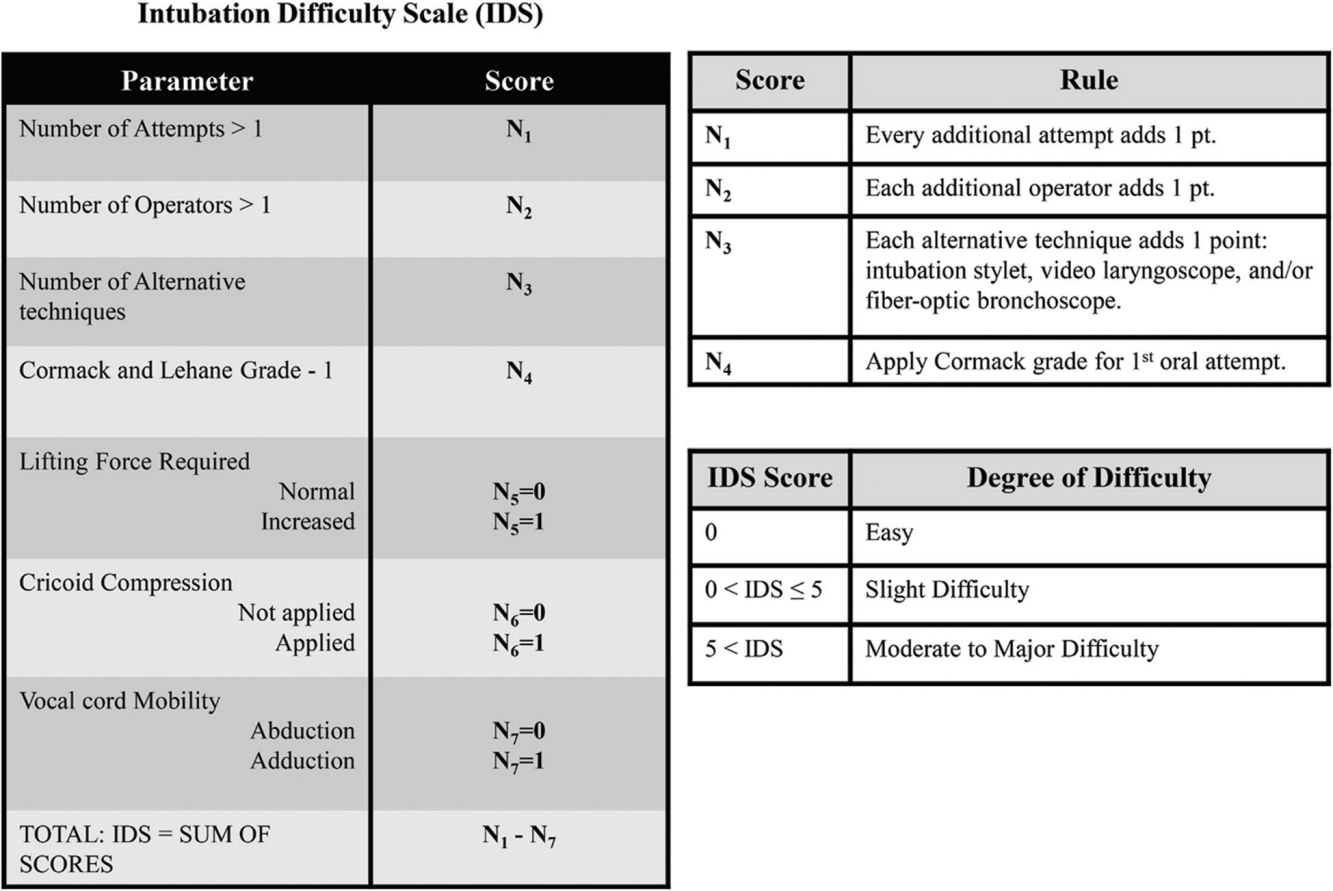
Intubation difficulty scale

After transport to the operating room, standard non-invasive monitoring of vital signs was started. Mouth aperture, Mallampati score, and SMD were measured again, just before anesthetic induction. In each hospital, the induction of general anesthesia and intubation were performed by a blinded anesthesiologist (clinical professor) who was unaware of patient allocation, and had more than 7 years of experience with anesthesia. After denitrogenation with 100% oxygen, propofol 1.5–2 mg/kg was intravenously administered, and target-controlled infusion of remifentanil with 4 ng/ml effect site concentration was started to inhibit hemodynamic responses to tracheal intubation. After patient consciousness and eyelash reflex were lost, rocuronium 0.6 mg/kg was administered, and facial mask ventilation was performed. Muscle relaxation was assessed using train-of-four stimuli every 10 s. Following the disappearance of T1 of train-of-four stimuli, orotracheal intubation was performed using a direct laryngoscope with Macintosh blade. During intubation, the anesthesiologist placed the laryngoscope into the mouth and raised the blade up and away from the patient to obtain a clear view of the glottis. The subjective necessity of increased lifting force compared to routine practice, and the Cormack-Lehane grade, were checked. To facilitate intubation, laryngeal compression (backward, upward, and rightward pressure) was sometimes applied by an assistant [20]. If we could not obtain an adequate view, orotracheal intubation was attempted with alternative techniques including intubation stylet, video laryngoscope, and/or fiber-optic bronchoscope. If the first anesthesiologist failed to intubate within 3 attempts, the fourth trial was performed by another senior anesthesiologist [21]. The requirements for laryngeal compression or alternative techniques, the number of intubation attempts and additional anesthesiologists, and the intubation time from the end of mask ventilation to the passage of an orotracheal tube through the vocal cords, were recorded by an independent and blinded anesthetic nurse in each hospital. Intubation difficulty scale (IDS) was calculated based on these values (Fig. 2) [2, 22, 23].

After anesthetic induction, anesthetic depth was maintained using 1–1.5 minimum alveolar concentration inhalation (sevoflurane or desfluane), or target-controlled infusion of propofol at a 40–60 bispectral index, with remifentanil target-controlled infusion adjusted to maintain hemodynamic stability. At the end of the operation, neostigmine 0.03 mg/kg and glycopyrrolate 0.01 mg/kg were administered to reverse residual neuromuscular block. When the train-of-four ratio increased over 90% and sufficient spontaneous breathing was confirmed, oropharyngeal secretions were gently cleared by suction, and the endotracheal tube removed.

At the post anesthesia care unit of each hospital, 15 min after the extubation, injuries of oropharyngeal soft tissue including lip, gum, tongue, buccal mucosa, palate, and pharynx were examined using a penlight by a single blinded anesthesiologist who participated only in this measurement and had no other involvement in the study. Except in nasal surgery cases, the appearance of bloody secretions in the oropharyngeal suction before extubation was also interpreted as incidence of oropharyngeal injury.

### Statistical analysis

Continuous variables are expressed as mean (SD). Categorical variables are shown as number or percentage (%). The primary outcome was the Mallampati score before and after exercise. Secondary outcomes were as follows: mouth aperture size, SMD, Cormack-Lehane grade, intubation time, numbers of intubation attempts, number of anesthesiologists who attempted orotracheal intubation, number of alternative techniques required, increased lifting pressure or laryngeal compression, soft tissue injuries after using direct laryngoscope, and IDS. To compare continuous variables between control and exercise groups, we used the independent Student’s t-test. Differences between categorical variables were assessed using the chi-square or Fisher’s exact test between the two groups. For grading characteristic of Mallampati score and Cormack-Lehane grade, linear-by-linear association was performed to compare the trend between groups. To analyze within-group change between before and after the exercise therapy, a paired t-test was used. SPSS version 19.0 software (SPSS Inc., IBM, Chicago, IL, USA) was used for the statistical analysis. All reported *P*-values are two-sided, and a *P*-value < 0.05 was considered statistically significant.

### Sample size

In a pilot study of 20 patients (10 patients in each group), the effect size for Mallampati score was used to calculate the required sample size (number of patients). The Mallampati score indicates mouth aperture size relative to tongue size, and therefore reflects the adequacy of mouth opening during displacement of the tongue by the laryngoscope [3, 19, 24]. Mallampati score is one of the well-recognized predictive factors for the condition of orotracheal intubation, and a Mallampati score other than 1 was reported as one of the criteria for predicting the condition [3, 5]. The incidence of Mallampati scores of 1 after the exercise regimen were 15% for the control group, and 35% for the exercise group. Based on the results of the pilot study, a power analysis was performed with G*Power 3.1.2 (Heinrich-Heine University, Düsseldorf, Germany). This suggested that in each group, 70 patients were required for a power of 80%, a risk of 0.05 for a type-I error in two-tailed statistical analysis, and a dropout rate of 7.5%.

## Results

**Fig. 3:**
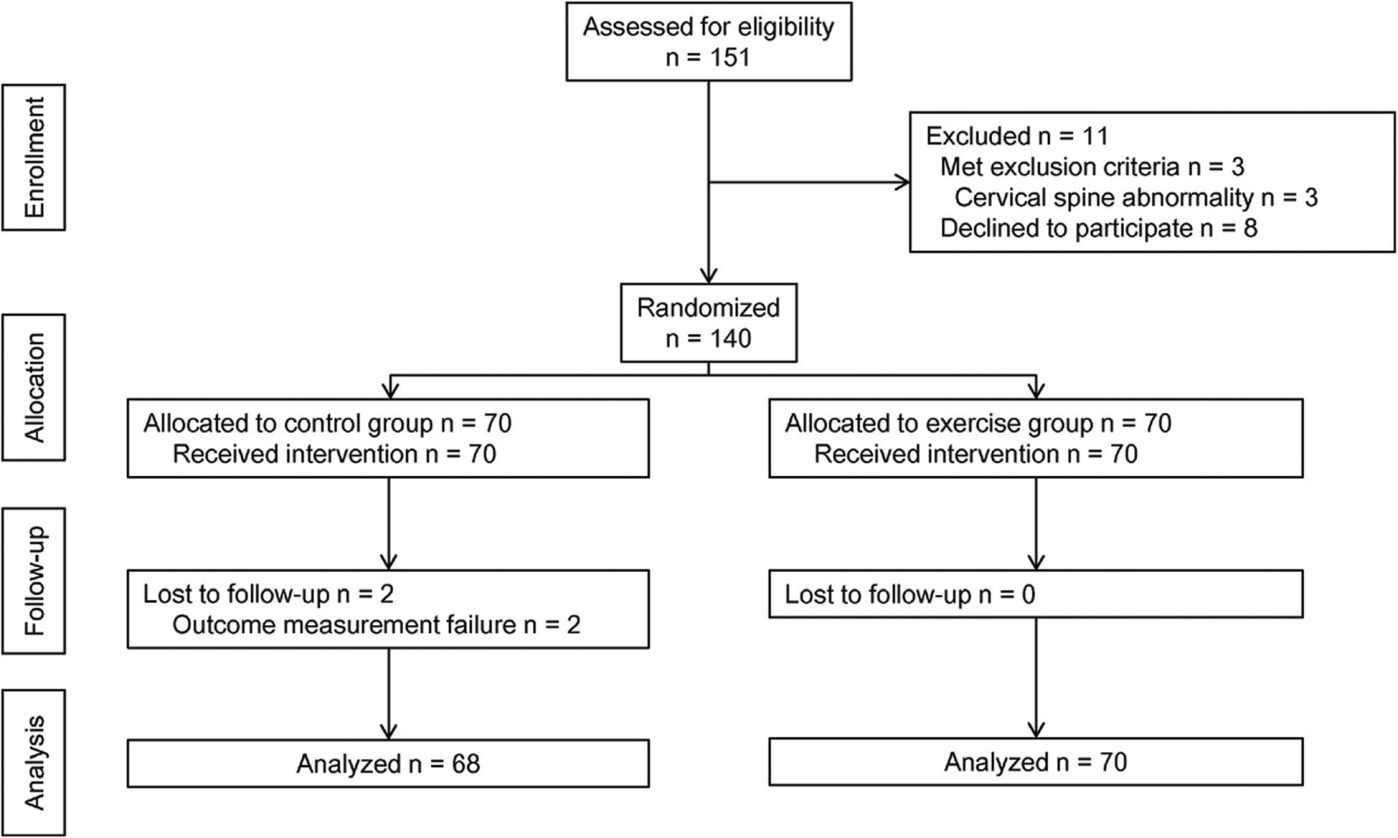
CONSORT diagram

Of the 151 eligible patients, 11 were excluded because 8 declined to participate, and 3 met the exclusion criteria for cervical pain. The remaining 140 patients were randomly allocated to one of the two study groups, with 70 patients per group. Two patients in the control group withdrew after the training intervention. Thus, 138 patients completed the study (number of patients in the control and exercise groups, 44 and 47 respectively in Seoul National University Bundang Hospital, and 24 and 23 in Jinju Gyeongsang National University Hospital; Fig. 3).

Patients’ baseline characteristics did not differ between groups, including mouth aperture size, SMD, pre-intervention Mallampati score, anesthesia, and surgery data affecting the intubation difficulty or the postoperative soft tissue assessment between the study groups (Tables 1 and 2).

**Table 1:** Patients’ characteristics, anesthesia, and operational data

	Control (*n* = 68)	Exercise (*n* = 70)
Age (years)	49.5 ± 11.6	50.6 ± 10.6
Male sex	32 (47.1%)	32 (45.7%)
Height (cm)	165.1 ± 9.1	163.9 ± 9.0
Weight (kg)	65.2 ± 11.8	68.0 ± 11.4
ASA class (I/II)	27 (39.7%) / 41 (60.3%)	23 (32.9%) / 47 (67.1%)
Thyromental distance (mm)	91.1 ± 9.8	91.8 ± 10.2
Buck teeth	1 (1.5%)	3 (4.3%)
Induction dose (propofol, mg)	112.6 ± 13.2	114.9 ± 10.5
Anesthetic time (min)	123.6 ± 74.7	139.9 ± 82.4
Operation Position (supine/prone/lateral decubitus)	66/2/0	69/0/1
Nasal surgery	4 (5.9%)	9 (12.9%)

Continuous values are shown as mean ± SD. Categorical variables are expressed as patient numbers (%) or numbers

*ASA* American society of anesthesiologists

**Table 2:** Mouth aperture size, SMD, and Mallampati score

	Control (*n* = 68)		Exercise (*n* = 70)		*P*_1_^a^	*P*_2_^b^	Estimated difference^b^ (95% CI)	*P*_3_^c^	Odds ratio^c^(95% CI)	*P*_4_^d^	Estimated difference^d^ (95% CI)
	Before	After	Before	After							
Mouth aperture size (mm)	49.3 ± 6.4	49.0 ± 7.5	48.9 ± 5.0	51.3 ± 6.4	0.675	0.042	−2.4(−4.8 to − 0.1)			< 0.001	−2.5(−3.7 to −1.2)
SMD (mm)	183.6 ± 16.0	183.6 ± 16.0	181.8 ± 16.0	187.3 ± 15.8	0.505	0.175	−3.7(− 9.0 to 1.7)			< 0.001	−5.5(− 6.4 to − 4.6)
Mallampati score (I/II/III/IV)	18/22/14/14	18/23/14/13	21/19/8/22	30/19/15/6	0.647	0.039		0.043	2.1 (1.0 to 4.3)		

Continuous values are shown as mean ± SD. Categorical variables are expressed as numbers

*SMD* Sternomental distance

^a^Comparisons of baseline (before the intervention) mouth aperture, SMD, and Mallampati score between the two groups using Student’s t-test (mouth aperture and SMD) and linear-by-linear association (Mallampati score)

^b^Comparisons of mouth aperture, SMD, and Mallampati score after the intervention, between the two groups using Student’s t-test (mouth aperture and SMD) and linear-by-linear association (Mallampati score)

^c^Comparison of the incidence of Mallampati score 1 after intervention between the two groups using chi-square test

^d^Comparisons of mouth aperture and SMD between before and after the intervention within the exercise group using paired t-test

At anesthetic induction, there was a significant difference in Mallampati score between the two groups (*P* = 0.039) and the incidence of Mallampati scores of 1 was higher in the exercise than in the control group (2.1 (1.0–4.3), odds ratio (95% CI); *P* = 0.043; Table 2). Mouth opening was after the intervention greater in the exercise group than in the control group (*P* = 0.042), however, SMD was indistinguishable between the control and exercise groups (*P* = 0.175; Table 2). Analysis of pre- to post-exercise therapy changes within the exercise group showed that the exercise intervention increased mouth aperture size and SMD (*P* < 0.001; Table 2).

The incidences of increased lifting force and laryngeal compression required to obtain the intubation pathway were lower in the exercise than in the control group (*P* = 0.034 and 0.027, respectively; Table 3). All other variables of IDS, the IDS score itself, and IDS difficulty group did not differ significantly between the two groups (Table 3). In both groups, there was no case in which an additional anesthesiologist was requested to attempt tracheal intubation (Table 3). Compared to the control group, the exercise group showed shorter intubation time and fewer soft tissue injuries (*P* = 0.032 and 0.009, respectively; Table 3). There were 58 patients with a baseline Mallampati score III or IV, who were deemed to show smaller mouth opening during tracheal intubation. In the subgroup analysis performed for those patients, there was a significant difference in the IDS grade between the control and exercise groups (*P* = 0.029; Table 4). The Cormack-Lehane grade did not significantly differ between the groups in the subgroup comparison (Table 4).

**Table 3:** Intubation difficulty, intubation time, and soft tissue injury

	Control(*n* = 68)	Exercise(*n* = 70)	*P* ^a^	Estimated difference (95% CI)	Odds ratio(95% CI)
Number of attempts	1.1 ± 0.4	1.1 ± 0.2	0.253	0.1(− 0.0 to 0.2)	
Additional operator	0 (0%)	0 (0%)			
Alternative techniques required	8 (11.8%)	4 (5.7%)	0.207		0.5 (0.1 to 1.6)
Cormack-Lehane grade (I/II/IIIa/IIIb)	42/20/2/4	45/19/3/3	0.746		
Increased lifting force	28 (41.2%)	17 (24.3%)	0.034		0.5 (0.2 to 1.0)
Laryngeal pressure required	24 (35.3%)	13 (18.6%)	0.027		0.4 (0.2 to 0.9)
Vocal cord mobility (adduction)	2 (2.9%)	0 (0%)	0.241		
IDS	1.6 ± 2.1	1.0 ± 1.7	0.086	0.6(−0.1 to 1.2)	
IDS group (easy/slight difficulty/moderate to major difficulty)	31/31/6	44/23/3	0.112		
Intubation time (s)	18.8 ± 23.1	12.5 ± 5.4	0.032	6.3 (0.5 to 12.0)	
Soft tissue injury	14 (20.6%)	4 (5.7%)	0.009		0.2 (0.1 to 0.8)

Continuous values are shown as mean ± SD. Categorical variables are expressed as patient numbers (%) or numbers

*IDS* Intubation difficulty scale

^a^Comparisons between the two groups using Student’s t-test (continuous variables), chi-square or Fisher’s exact test (categorical variables), and linear-by-linear association (Cormack-Lehane grade)

**Table 4:** Intubation difficulty among the patients with baseline Mallampati score III or IV

	Control (*n* = 28)	Exercise (*n* = 30)	*P* ^a^
Cormack-Lehane grade (I/II/IIIa/IIIb)	11/12/2/3	19/9/0/2	0.098
IDS group (easy/slight difficulty/moderate to major difficulty)	8/15/5	19/9/2	0.029

Categorical variables are expressed as patient numbers

*IDS* Intubation difficulty scale

^a^Comparisons between the two groups using linear-by-linear association (Cormack-Lehane grade) and chi-square test (IDS group)

## Discussion

The beneficial effects of stretching or massage on different joints have been studied for various therapeutic applications [12–18]. A variety of exercise-treatments have been demonstrated to relieve clinical symptoms and restore range of motion in joint disorders, including osteoarthritis and rheumatoid arthritis [11, 25–28]. The exercise regimen of our study protocol included active and passive stretching of jaw and neck muscles. A brief stretching session potentially enhances joint flexibility and alleviates muscle stiffness [12, 13, 18]. In a previous study of knee joint exercise, 4 sets of stretching for 20 s each produced a decrease in passive hamstring stiffness, and improved joint range of motion [12]. Massage has been also reported to reduce muscle stiffness [15, 16]. In our protocol, the patients in the exercise group performed masseter muscle massage at the beginning and at the end of the exercise therapy. To our knowledge, this study is the first quantification of the effect of a simple exercise regimen on mouth opening and neck extension during anesthetic induction.

During induction of general anesthesia, orotracheal intubation is usually performed under adequate muscle relaxation that can be achieved by administration of a neuromuscular blocking agent. However, a neuromuscular blocking agent exerts its effects at the neuromuscular junction. It does not modulate the mechanical properties of joints but rather interferes with neural drive to muscle, thereby blocking contraction [29]. Joint flexibility or stiffness is dependent not only upon the contraction of joint muscles, but also on the properties of connective tissue and ligaments [30–33]. Therefore, the enhancement of mouth opening and neck mobility by pre-anesthetic exercise could be maintained even after neuromuscular blocking agent injection. Furthermore, it was reported that general anesthesia employing muscle relaxants did not necessarily increase the passive range of motion of joints [30].

Although SMD examined just before anesthetic induction was comparable between the two groups, the clear differences before and after the regimen within the exercise group showed the definite beneficial effects of our exercise therapy on mouth opening and neck extension. The exercise group showed larger mouth opening and enhanced Mallampati scores, as we had anticipated (Table 2). Strictly speaking, the mouth opening and SMD evaluated in our study are induced by active muscle contraction, and therefore, are different from the passive processes during the orotracheal intubation using direct laryngoscopy. However, improvement of joint flexibility and muscle stiffness by the exercise therapy led to a substantial treatment effect on the measured values (Table 2). The objective increase of mouth opening and neck extension seems to have influenced the clinical differences between the two groups (Table 3).

The IDS comprehensively scores intubation difficulty, because it combines 7 measurable variables (Fig. 2) [2, 22, 34]. In our study, the incidences of increased lifting force and laryngeal compression were lower in the exercise group than in the control group (Table 3). The enhanced joint range of motion and reduced muscle stiffness seem to induce the discrepancies. Conversely, the Cormack-Lehane grade did not differ between the two study groups. This suggests that comparable visualization could be obtained by increasing lifting force or laryngeal compression in the control group. The total IDS scores were also indistinguishable between the two groups (Table 3). However, the additional force and pressure necessary for patients in the control group increased intubation time and caused more frequent oropharyngeal soft tissue injury (Table 3). For the patients with a baseline Mallampati score III or IV, the pre-anesthetic intervention was associated with less intubation difficulty during anesthetic induction, which highlighted the value of the brief regimen (Table 4). The exercise intervention in our protocol was intended to increase the range of motion in jaw and neck joints by enhancing joint flexibility and reducing muscle stiffness, which seemed to be more effective for improving intubation conditions in the patients who had a relatively small mouth aperture size.

In this study, we excluded patients with temporomandibular joint disorders or cervical spine diseases. Unless active movement is impossible or would induce neurologic symptoms, patients at high risk for intubation difficulties should be able to manage the exercise and might benefit from this therapy, which would be clinically valuable. However, since this study was the first clinical trial demonstrating the effect of pre-anesthetic exercise, we excluded those patients to avoid conditions where proper stretching was not possible due to joint pain, and to maintain uniformity in the 5 min intervention. Long-time and steady exercise program might be more helpful for this cohort. Further studies would be necessary to identify the effect of preoperative exercise regimen on intubation conditions in patients with an increased risk for difficult intubation.

Some limitations of this study should be mentioned. First, the IDS incorporates some subjective parameters: the perception of lifting force and the application of alternative techniques or laryngeal compression may vary among anesthesiologists. It was also proposed that the intubation difficulty should be evaluated with simple and objective parameters such as Cormack-Lehane grade and intubation time, rather than IDS [35]. However, in our protocol, the orotracheal intubation was performed by a single blinded anesthesiologist in each hospital to exclude any possible inter-assessor bias and increase the reliability of the IDS scoring. Furthermore, there was a significant difference in the intubation time between the control group and the exercise group. However, inter-rater variability still existed between the two hospitals. To minimize the bias, each investigator tried to perform every measurement or intervention uniformly and strictly according to the study protocol standardized in advance. Second, the duration from the end of exercise regimen to the anesthetic induction was not controlled uniformly. In order to maintain the blindness, the intervention was performed at the reception, not in the OR. The time for transportation to the operating room and preparation for general anesthesia was not strictly constant. However, the delay did not extend beyond 5 min in both hospitals, and the exercise significantly improved mouth opening and neck extension during anesthetic induction. In a previous report, simple stretching for less than 2 min alleviated passive hamstring stiffness for 20 min [12]. Third, Mallampati score, the primary outcome of the study, has been criticized for its low predictive value for difficult intubation [36–38]. However, we did not hypothesize that the pre-anesthetic regimen would reduce intubation difficulty, rather, we anticipated that the exercise therapy we describe would increase range of motions of mouth opening and neck mobility during the anesthetic induction, thereby possibly reducing intubation time and tissue injuries associated with intubation (Table 3). We utilized the Mallampati score to assess the clinical effects of the exercises over other simple lengths. Fourth, penlight examination might miss injuries in the deep hypopharyngeal or laryngeal structures. Although the appearance of bloody secretions in the oropharyngeal suction could help identify the injuries, this was also not an objective and accurate measurement. To reduce inter-rater variability and to maintain consistency of the test, a single blinded anesthesiologist in each hospital performed the examinations. Fifth, patients in our study showed a higher proportion of Mallampati score IV, compared to previous studies [39, 40]. No phonation during the measurement and the relatively small number of patients might have influenced the distribution of our findings. However, in previous research, the distribution of Mallampti scores also varied according to the patients’ characteristics [41]; the most important outcome of this study was that the exercise regimen significantly changed it.

## Conclusion

A 5 min pre-anesthetic exercise session facilitated mouth opening and neck extension during orotracheal intubation, improving intubation conditions and enabling faster intubation with less injury to oropharyngeal soft tissue. The clinical effect of the brief regimen seemed to be more significant in patients with higher Mallampati scores. Our results suggest that incorporation of such therapy into pre-operative procedures may be beneficial for patients. Patients undergoing general anesthesia can easily perform the simple exercise during the waiting time before anesthetic induction.

## Data Availability

The dataset generated and analyzed during the current study is available from the corresponding author on reasonable request.

## Abbreviations

ASA: American Society of Anesthesiologists
ISD: Intubation difficulty scale (IDS)
SMD: Sternomental distance
